# Effects of Sling-Suspension-Based Active Shoulder Joint Exercise on Shoulder Joint Subluxation, Pain, Muscle Strength, and Upper Limb Function in Patients with Subacute Stroke

**DOI:** 10.3390/medicina60081350

**Published:** 2024-08-20

**Authors:** Young-Jun Kim, Jungae An, Byoung-Hee Lee

**Affiliations:** 1 Graduate School of Physical Therapy, Sahmyook University, Seoul 01795, Republic of Korea; youngjun6507@hanmail.net; 2 Department of Physical Therapy, Sahmyook University, Seoul 01795, Republic of Korea; ajapt@syu.ac.kr

**Keywords:** stroke, sling suspension, subluxation, upper extremity function

## Abstract

*Background and Objectives*: We investigated the effects of sling-suspension-based active shoulder joint exercise training on shoulder joint subluxation, pain, muscle strength, and upper extremity function in patients with subacute stroke. *Materials and Methods*: Twenty-eight patients with subacute stroke were randomly assigned to either the sling-suspension-based active shoulder joint exercise (SASE) group (*n* = 14) or the motorized upper extremity exercise (MUEE) group (*n* = 14). The SASE group actively performed shoulder joint flexion, extension, abduction, adduction, external and internal rotation, and horizontal abduction and adduction using a sling suspension system, whereas the MUEE group underwent an exercise program using a motorized upper extremity exercise machine. All participants underwent a 4-week intervention with 30 min of exercise once a day for 5 days a week. Additionally, both groups received general physical therapy and functional electrical stimulation for 30 min twice a day for 5 days a week. Shoulder joint subluxation was measured by radiographic examination before and after training, and pain was evaluated in the splenius, upper trapezius, and infraspinatus muscles using pressure parameters. In addition, a manual muscle tester was used to assess the muscle strength of the shoulder joint flexors, extensors, abductors, adductors, and external and internal rotators, and the Fugl–Mayer Assessment (FMA) and Manual Functional Test (MFT) were used to evaluate upper extremity function. *Results*: A significant group–time interaction was observed for pain, with F-values of *F*(1, 26) = 7.470, *p* < 0.011 for the splenius and *F*(1, 26) = 9.623, *p* < 0.005 for the upper trapezius. A significant time–group interaction was observed for the muscle strength of the shoulder, with F-values of *F*(1, 26) = 13.211, *p* < 0.001; *F*(1, 26) = 4.974, *p* = 0.035 and *F*(1, 26) = 9.674, *p* = 0.004 for flexors, abductors, and external rotators, respectively. A significant time–group interaction was observed in the FMA, with F-values of *F*(1, 26) = 13.243, *p* < 0.001. When comparing the interaction effects between time and group for MFT scores, a significant difference was observed, with F-values of *F*(1, 26) = 32.386, *p* < 0.001. *Conclusions*: This study confirmed that sling-suspension-based active shoulder joint exercises are effective in improving shoulder joint subluxation, pain, muscle strength, and upper extremity function in patients with subacute stroke.

## 1. Introduction

Musculoskeletal impairment may occur after stroke and includes shoulder subluxation, pain, and contractures. Shoulder subluxation is one of the most common complications, with an incidence rate of 80% [1]. Approximately 17% of patients experience shoulder pain within 1 week after the onset of stroke, with 55, 87, and 75% experiencing pain within 2 weeks, after 2 months, and within 1 year, respectively. Shoulder subluxation typically occurs in the early stages of stroke, during the flaccid stage; however, it can also be compounded by the onset of rigidity or severe rigidity. This can persist until the chronic stage if upper limb function does not recover [2]. Shoulder subluxation is defined as the dynamic alteration of the shoulder joint that facilitates the gap between the shoulder acromion and the head of the humerus [3]. During the flaccid stage of stroke, the body tends to tilt or shorten towards the paralyzed side. Some patients with stroke experience impaired balance when the affected arm shakes [4]. Shoulder subluxation is an important cause of secondary complications that affect the ligaments, muscles, nerves, blood vessels, and joint capsules around the shoulder. This leads to restricted range of motion and pain in the shoulder joint, thereby delaying neurological recovery [5,6]. Paralysis or weakness of the muscles around the shoulder and initial subluxation owing to inaccurate alignment of the shoulder joint do not initially cause pain. However, continuous inaccurate alignment owing to traction exposes the joint to dynamic pressure and gravity, leading to the onset of pain. Incorrect positioning of the joint can result in impingement syndrome, tendinitis, adhesive capsulitis, and damage to the brachial plexus [7,8]. Consequently, shoulder subluxation restricts the ability of patients with stroke to perform activities of daily living that involve the use of their arms, making it challenging to achieve rehabilitation goals and impeding functional recovery. Therefore, active prevention of shoulder subluxation during stroke rehabilitation and pain reduction are crucial. Methods for preventing and reducing shoulder subluxation include the use of a sling [9], electrical stimulation therapy [10], arm supports and wheelchair tables [11], and taping techniques [12]. Wearing a sling helped postural adaptation, acting as a feedback mechanism for re-educating patients about their perception of their arm [11]. It also helps improve the forward bending of the trunk during walking.

Furthermore, sling suspension systems allow exercise to be performed with reduced friction against the floor, minimizing the resistance imposed on movement by eliminating the gravitational force obtained from hanging on a suspended rope [11]. Sling suspension systems can induce gradual muscle contractions in patients with stroke, and resistance intensity and direction are easily adjustable [13]. Active exercise is most suitable and therapeutic approaches include relaxation exercises, stabilizing muscle training, increasing range of motion, traction, sensorimotor integration training, muscle strengthening, and endurance exercises [14].

To date, research on sling suspension systems targeting patients with stroke has predominantly focused on trunk stabilization and strengthening exercises or exercises for the lower limbs. There is a lack of research specifically addressing shoulder subluxation and upper limb function in patients with stroke. In the rehabilitation of stroke patients with shoulder subluxation, tissue damage and pain caused by subluxation affect upper limb function. Although previous studies reported the effects of sling-suspension-system-based active exercise on subluxation distance, proprioception, and upper extremity function in stroke patients, the effects on pain and muscle strength around the shoulder joint in stroke patients were not verified [15]. Therefore, this study investigated the effects of sling-suspension-based active shoulder joint exercises on shoulder subluxation, pain, muscle strength, and upper limb function in patients with acute stroke and shoulder subluxation.

## 2. Materials and Methods

### 2.1. Participants

This study included 40 adult patients with acute-stage stroke admitted to D Hospital, Daejeon Metropolitan City, South Korea. Before recruiting participants for this study, we performed a power analysis using G*Power version 3.1.9.7 (Franz Faul, University Kiel, Kiel, Germany, 2020) with ANOVA (repeated measures, with in-between interaction). An effect size f of 0.25 was obtained for all the outcome measures, with an α-error probability of 0.05, to minimize type 1-β error probability of 80%, with two groups and four measurements. As the estimated target sample size was 32, we recruited 45 participants who underwent physical therapy post-stroke.

The inclusion criteria for this study were as follows: a diagnosis of hemiparesis due to stroke within 3–6 months of stroke onset, a gap greater than one finger breadth between the shoulder acromion and the head of the humerus, absence of orthopedic conditions affecting the shoulder joint, a Mini-Mental State Examination, Korean (MMSE-K), score of 21 or higher indicating no communication difficulties, shoulder flexor and abductor muscle strength ranging from P+ to F−, and ability to sit independently for at least 20 min.

The following exclusion criteria applied: pain with no symptoms of subluxation, history of shoulder joint pathology before the onset of stroke, and fractures or trauma involving the paralyzed side of the shoulder joint.

All participants signed a consent form after the purpose of this study and details of the procedures involved were explained. This study was approved by the Sahmyook University Institutional Review Board (approval number: 2-1040781-A-N-012021057HR) and Clinical Research Information Service (KCT0006535). The participants fully understood the objectives and procedures used in the study. The study adhered to the ethical principles of the Declaration of Helsinki.

### 2.2. Experimental Procedure

Approval for access to patient medical records was obtained prior to experimentation. The following patient data were collected: past medical history, present symptoms, surgical dates, and general characteristics such as age, height, weight, duration of illness, affected limb, cause of onset, and MMSE-K scores. Participants underwent a pre-experimental medical examination by a physician, and their medical history and other characteristics, including orthopedic or neurological examination findings, were recorded. Shoulder subluxation was measured using radiographic examinations, pain levels were assessed using a pressure algometer, and muscle strength was evaluated using a portable dynamometer. Upper limb function was assessed using the Fugl–Meyer Assessment (FMA) and Manual Function Tests.

To minimize errors related to group selection and reduce bias, the Research Randomizer program (http://www.randomizer.org/, accessed on 15 August 2021) was used to randomly divide the participants into two groups. These groups were then assigned to the therapists to minimize bias and ensure unbiased experimentation. The sling-suspension-based active shoulder joint exercise (SASE) group underwent sling-suspension-based active shoulder joint exercises. Active shoulder joint exercises using the sling suspension system were performed for 30 min once daily, 5 days a week. The motorized upper extremity exercise (MUEE) group underwent electric upper limb exercises and utilized electric upper limb exercise equipment for 30 min once daily, 5 days a week. Both groups underwent general physical therapy and functional electrical stimulation (FES) twice daily for 30 min per session, 5 days a week, for a total intervention period of 4 weeks. After 4 weeks, shoulder subluxation, pain levels, muscle strength, and upper limb function were evaluated using the same measurement tools.

**Figure 1:**
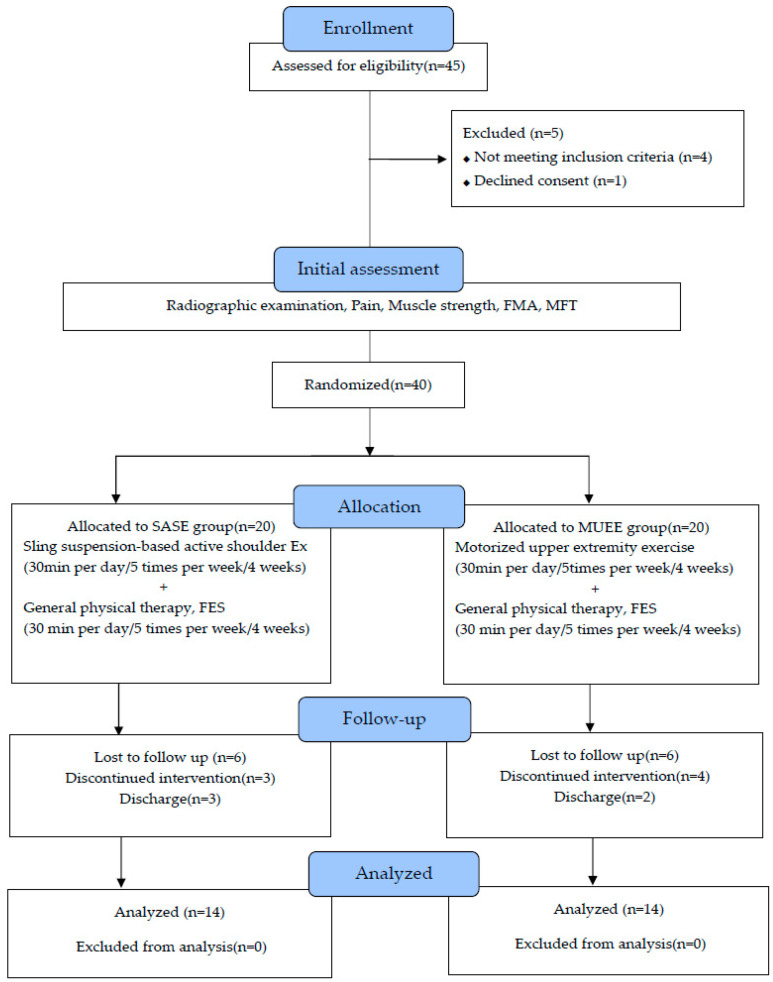
Flow diagram of the study participants.

Participants with a participation rate of <90% were excluded from the final study population. Following the exclusion of 12 individuals (6 from each group) due to hospital discharge (3 from the sling-suspension-based active shoulder joint exercise group and 2 from the motorized upper extremity exercise group) or personal reasons (3 from the sling-suspension-based active shoulder joint exercise group and 4 from the motorized upper extremity exercise group), a total of 28 participants were included in the final analysis (Figure 1).

The intervention was administered by physical therapists with at least 5 years of experience, while the measurements were conducted by physical therapists with over 3 years of experience. The physical therapists were knowledgeable about the characteristics of acute stroke and potential issues that may arise during the research process. Additionally, physical therapists received training on the use of measurement equipment 1 week before starting treatment to minimize error margins. Measurements were conducted by the same physical therapist before and after the experiment, and the assessors were blinded to the groups.

### 2.3. Training Program

#### 2.3.1. The Sling-Suspension-Based System Active Shoulder Joint Exercise

**Figure 2:**
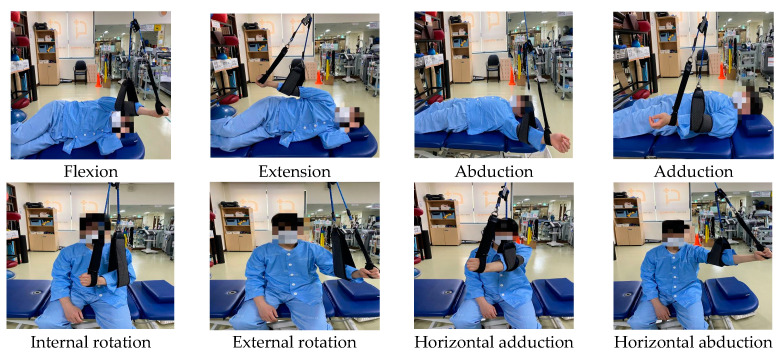
The sling-suspension-based active shoulder joint exercise training.

This study used the sling suspension system (PRO SLING SYSTEM, Promedi, Wonju, Republic of Korea, 2018) for active shoulder joint exercises. The training program consisted of four components based on previous studies, but the training time and weight of resistance were applied differently [15]. Horizontal abduction–adduction exercises were performed with the arm in a flexed position while seated in a chair. The internal–external rotation exercise of the shoulder joint was performed with the arm flexed at 90° while seated in a chair. The abduction–adduction exercise was performed with the arm in a relaxed position while lying flat on the back. The flexion–extension exercise was performed with the arm in a relaxed position and the affected side facing downward while lying on one side. The exercises followed the principle of active movement within the available range of joint motion. A total of 5 sets of 15 repetitions of each exercise were performed, totaling 75 repetitions within a 7 min period. After completing all the exercises, the next exercise program was initiated. Thus, all four movements were performed over 30 min. The intensity of the exercise was adjusted by the position of the suspension point and by using sandbags weighing 0.5–2 kg to set the resistance level. The suspension point was positioned vertically above the shoulder joint or inward towards the body to ensure that the head of the humerus fitted snugly Into the glenoid fossa. Additionally, straps were used during the exercise to maintain the arm in a flexed position and prevent flexion of the elbow joint. The intervention was conducted by the same physical therapist with over 5 years of experience, after completing sling education training (Figure 2).

#### 2.3.2. Motorized Upper Extremity Exercise

The electric upper limb exercise equipment used in this study was the MotorCross MC-3 (Promedi, Wonju, Republic of Korea, 2018). This equipment is designed for patients with upper limb disabilities and allows both passive and active exercises. The speed ranges from 0 to 60 revolutions per minute (rpm) and the motor force levels range from 0 to 12. In addition, it allows for direction control in both the forward and backward directions. Passive exercises were conducted in the forward direction at 15 rpm with a motor force of 4. These exercises were performed once daily for 30 min, five times a week, for 4 weeks.

#### 2.3.3. General Physical Therapy

The phrase “one-on-one treatment by a physical therapist with interventions based on the developmental stages of the central nervous system” refers to a therapeutic approach tailored to the neurological development of the patient. Interventions include Bobath neurodevelopmental therapy, proprioceptive neuromuscular facilitation, joint range-of-motion exercises, stretching, and strengthening exercises for the upper limbs, exercise control and coordination training, functional mat training, and gait training. All study participants underwent general physical therapy twice a day for 30 min per session, five times a week, for 4 weeks.

#### 2.3.4. Outcome Measures

Radiographic examinations were used to measure shoulder subluxation in patients with stroke. Radiographic imaging was conducted using shoulder anteroposterior projections to capture images of the shoulder joint from both front and back. Images were taken with the participant seated in a chair with both upper limbs hanging naturally, without any support. The central ray was angled perpendicular to the cassette surface and directed towards the inner corner of the humeral head. Radiographic imaging was performed by the same radiographer wearing radiation-protection aprons. Subluxation was measured at three points: the center of the glenoid fossa, center of the humeral head, and the most inferior–lateral point of the acromion. These points were used as references as described by [16]. The center of the glenoid fossa was defined as the point bisecting the two lines measured from the maximum length and width of the glenoid fossa, whereas the center of the humeral head was defined as the point bisecting the line with the greatest diameter measured from the humeral head. Inferior humeral head displacement was defined as the distance between the center of the humeral head and the most inferior lateral point of the acromion. Lateral humeral head displacement was defined as the distance between the centers of the humeral head and glenoid fossa. The radiographic images were interpreted by a radiologist specializing in diagnostic imaging. The average value from three separate imaging sessions was used. A positive value obtained by subtracting the distance on the affected side from that on the unaffected side indicated the presence of subluxation [17].

This study used a pressure algometer (Wagner Force Ten-FDX; Wagner Instrument, Greenwich, CT, USA, 2010) to assess the degree of pain in the shoulder joint area of patients with stroke. Very high intra-rater reliability (r = 0.85) and inter-rater reliability (r = 0.85) of the pressure algometer have been reported [18]. The muscle measurement sites were as follows: the upper trapezius (UTZ) was measured 1 cm lateral to the fourth cervical vertebra; the deltoid was measured at the midpoint between the spinous process of the seventh cervical vertebra and the acromion; and the infraspinatus (IST) was measured at the midpoint between the lower angle of the scapula and the lower corner of the axillary fold. During measurement, the patient was seated comfortably in a chair. The pressure algometer was positioned vertically at the measurement site and pressure was gradually applied at a rate of 1 kg/s. The patient was instructed to say “stop” when they began to feel pain or discomfort. A total of three measurements were taken, and the average value was used. All the measurements were performed by the same examiner [19].

A manual muscle tester device (Model 01163, Lafayette instruments, Lafayette, IN, USA, 2003) was used to measure the flexors and extensors, abductors and adductors, and internal and external rotators of the affected shoulder joint. The intra-rater reliability of the manual muscle testing device range was r = 0.84–0.99, while the inter-rater reliability range was r = 0.84–0.94, and the test–retest reliability range was r = 0.98–0.99 [20]. Muscle strength was measured by performing maximal voluntary contraction for 5 s at the midpoint of the maximum active range of motion. The measurement posture was consistent with each exercise posture and the average value was used after three measurements. All measurements were performed by the same examiner.

In this study, the FMA and the Manual Function Test (MFT) were used to evaluate upper limb function. The FMA consists of six items—motor function, balance, sensation, joint range of motion, and pain—and is a quantitative assessment tool for evaluating the functional recovery of patients poststroke. Motor function was scored out of 66 points for the upper limbs and 34 points for the lower limbs, with the performance of each item scored on a 3-point ordinal scale, where 0 = unable to perform, 1 = partially able to perform, and 2 = fully able to perform. A higher score indicates better function. An intra-rater reliability of r = 0.99 has been reported, with an inter-rater reliability range of r = 0.98–0.99 and a high correlation of r = 0.94 between the upper limb motor function assessment and the validity assessment [21]. The MFT is an assessment tool for measuring motor skills and upper limb function in patients with stroke. It consists of eight items: four for upper limb movements, two for grasping, and two for finger dexterity. The scores range from 0 (severe impairment) to 32 (full functional ability). A higher score indicates better function. An inter-rater reliability of r = 0.95 and intra-rater reliability above r = 0.95 have been reported [22].

#### 2.3.5. Data Analysis

The analyses were conducted using SPSS ver. 28.0 (IBM, Armonk, NY, USA, 2019) program. The normality of the entire participant pool was assessed using the Shapiro–Wilk test, and normality was confirmed for the main variables. Skewness and kurtosis values were found to be less than 2 and 7, respectively, satisfying the assumption of normality [23]. Descriptive statistics were used to describe the general characteristics of the participants, and homogeneity tests conducted before the intervention showed a normal distribution. Independent *t*-tests were used to assess homogeneity between the two groups, paired *t*-tests were used to analyze pre- and postintervention differences within each group, and independent *t*-tests were used to compare differences between the groups. Repeated-measures analysis of variance was used to evaluate the interaction effects of group and time. The statistical significance level for all analyses was set at 0.05.

## 3. Results

### 3.1. Participant General Characteristics

Table 1 presents the participant’s general characteristics and homogeneity test results. At baseline, no significant differences were observed between the two groups.

### 3.2. Subluxation of the Shoulder Joint

When examining the differences within each group before and after intervention, the SASE group showed a significant decrease from 44.59 mm before intervention to 36.97 mm after intervention (*p* < 0.05), and the MUEE group also showed a significant decrease from 40.03 mm before intervention to 36.35 mm after intervention (*p* < 0.05).

The SASE group exhibited a significant decrease compared to the MUEE group (*p* < 0.05). A significant interaction effect was observed between the group and time, as indicated by the F-values *F*(1, 26) = 6.025, *p* < 0.021, and a significant time effect was observed with F-values of *F*(1, 26) = 49.578, *p* < 0.001. No significant group effects were observed (Table 2).

### 3.3. Pain

When examining the splenius, the SASE group showed a significant increase from 3.92 kgf before intervention to 5.84 kgf after intervention (*p* < 0.05), and the MUEE group also demonstrated a significant increase from 3.85 kgf before intervention to 5.31 kgf after intervention (*p* < 0.05). When comparing the groups, the SASE group exhibited a significant increase compared to the MUEE group (*p* < 0.05).

When examining the UTZ, the SASE group showed a significant increase from 5.30 kgf before intervention to 7.74 kgf after intervention (*p* < 0.05), and the MUEE group also demonstrated a significant increase from 5.36 kgf before intervention to 7.08 kgf after intervention (*p* < 0.05). When comparing the groups, the SASE group exhibited a significant increase compared to the MUEE group (*p* < 0.05).

When examining the IST, the SASE group showed a significant increase from 4.27 kgf before intervention to 6.06 kgf after intervention (*p* < 0.05), and the MUEE group also demonstrated a significant increase from 3.85 kgf before intervention to 5.47 kgf after intervention (*p* < 0.05).

A significant interaction between group and time was observed, with F-values of *F*(1, 26) = 7.470, *p* < 0.011 for the splenius and *F*(1, 26) = 9.623, *p* < 0.005 for the UTZ. When examining the time effect, significant differences were revealed, with F-values of *F*(1, 26) = 384.345, *p* < 0.001; *F*(1, 26) = 323.720, *p* < 0.001; and *F*(1, 26) = 451.001, *p* < 0.001 for the splenius, UTZ, and IST (Table 3), respectively.

### 3.4. Muscle Strength of the Shoulder Joint

The muscle strength of the shoulder joint showed a significant time–group interaction with F-values of *F*(1, 26) = 13.211, *p* < 0.001; *F*(1, 26) = 4.974, *p* = 0.035 and *F*(1, 26) = 9.674, *p* = 0.004 for flexors, abductors, and external rotators, respectively; a significant time effect was observed with F-values of *F*(1, 26) = 236.665, *p* < 0.001, *F*(1, 26) = 175.005, *p* < 0.001, *F*(1, 26) = 128.769, *p* < 0.001, *F*(1, 26) = 170.356, *p* < 0.001, *F*(1, 26) = 125.739, *p* < 0.001, and *F*(1, 26 = 232.829, *p* < 0.001 for flexors, extensors, abductors, adductors, external rotators, and internal rotators, respectively (Table 4).

### 3.5. Upper Limb Function

The comparison results of the upper limb function between the SASE and MUEE groups are shown in Table 5. A significant time–group interaction for the FMA was observed, with F-values of *F*(1, 26) = 13.243, *p* < 0.001; a significant time effect was observed, with an F-value of *F*(1, 26) = 262.785, *p* < 0.001; and a significant group effect was observed, with an F-value of *F*(1, 26) = 5.805, *p* < 0.023. The comparison of pre- and postintervention measurements for FMA revealed significant increases in both the SASE group (from 13.29 points before intervention to 19.71 points after intervention) and the MUEE group (from 9.64 points before intervention to 13.71 points after intervention) (*p* < 0.05).

When comparing the interaction effect between time and group for the MFT scores, a significant difference was observed, with F-values of *F*(1, 26) = 32.386, *p* < 0.001. Significant differences were observed over time, with F-values of *F*(1, 26) = 647.498, *p* < 0.001; however, no significant group effect was reported. Pre- and postintervention MFT measurements were compared revealing significant increases in both the SASE group (from 6.57 points before intervention to 11.07 points after intervention) and the MUEE group (from 10.79 points before intervention to 13.64 points after intervention) (*p* < 0.05).

## 4. Discussion

In patients with stroke, shoulder girdle muscles such as the deltoid and trapezius are unable to perform their proper function due to initial flaccid paralysis, which prevents the downward movement of the head of the humerus. Additionally, the occurrence of subluxation arises from the downward rotation of the scapula as the joint fossa points downward [24]. Finding the most effective method to realign shoulder joint subluxation in patients with stroke is crucial [25]. This study investigated the effects of sling-suspension- system-based active shoulder joint exercises on subluxation, pain, muscle strength, and upper limb function in patients with subacute stroke.

In this study, the distance of shoulder subluxation in the SASE group decreased by 7.61 from 44.59 mm before intervention to 36.97 mm after intervention, while in the MUEE group, it decreased by 3.68 from 40.03 mm before intervention to 36.35 mm after intervention. The decrease in the degree of shoulder subluxation was significant in both groups (*p* < 0.05). When comparing between groups, the SASE group exhibited a significant decrease compared to the MUEE group (*p* < 0.05) and the time–group interaction effect was also significant. The previous study [15] examined the impact of active shoulder joint exercises using a sling-suspension-based system for 30 min, five times a week, for 4 weeks, and reported a significant difference in shoulder subluxation distance; it decreased by 4.71 mm from 10.86 mm before intervention to 6.21 mm after intervention. This difference in the intervention’s effect on subluxation distance is related to the onset duration and baseline subluxation distance of the stroke patients who participated in both studies. The stroke patients who participated in this study had an onset duration of 3 to 4 months, which corresponds to a more acute stage compared to the 18 to 19 months onset duration observed in participants in previous studies. Shoulder pain in stroke survivors gradually increases; within 6 months after stroke onset, approximately 5–84% of patients experience shoulder pain and various sensory disturbances [26].

The achievements of our study, compared to similar studies [15], are the demonstrated effects on pain and muscle strength around the shoulder joint. The result of the pressure pain in the splenius, UTZ, and IST muscles was measured using a pressure algometer in this study. The comparison of groups, splenius, and UTZ showed a statistical difference. A significant group–time interaction was also found in splenius and UTZ. Exercises using sling-suspension-based systems generally aim to improve muscle strength and endurance along with neuromuscular re-education. Therefore, the sling-suspension-based system was primarily developed to focus on stabilization exercises. They offer the advantage of being able to customize exercise programs according to individual fitness levels [27]. The reduction in shoulder joint pain is more closely correlated with the increase in muscle activity than with the degree of shoulder joint subluxation [28]. We consider that the sling-suspension-based active shoulder joint exercises had the activation of the rotator cuff and shoulder muscles involved in stabilizing the scapula compared to the exercises using the motorized upper limb exercise device. Furthermore, stabilization of the scapula contributes to maintaining the shoulder joint in the proper position [29,30], reducing collisions and instability between the scapula and shoulder joint, which we believe was more effective in reducing pain. In this study, no significant difference was observed in the level of pain in the IST muscle between the SASE group and the MUEE group. This finding may be explained by the fact that in the early stages of severe muscle weakness following stroke onset, the muscles involved in internal rotation of the shoulder joint are more predominant than those involved in external rotation. Consequently, the activity of the IST, which acts on the external rotation of the shoulder joint, may be lower than that of the muscles involved in internal rotation.

Although the splenius and UTZ were effective for pressure pain threshold, muscle strength tests showed different results. Using a dynamometer, we measured the strength of the shoulder flexors, extensors, abductors, adductors, and external and internal rotators. The muscle strength of the shoulder flexors, abductors, and external rotators showed a significant improvement in the SASE group than the MUEE group. The time–group interaction also presented significance in shoulder flexors, abductors, and external rotators. It has been reported that shoulder subluxation caused by acute stroke is reduced when the dynamic control ability of the scapula is promoted by selectively strengthening the rotator cuff and trapezius muscle according to the correct alignment of the shoulder joint complex [31,32]. The SASE group used a sling-suspension-based system to adjust the gravitational force applied to the shoulder joint according to muscle strength, enabling active movement in supine, side-lying, and seated positions; gradually increasing exercise intensity in response to muscle strength recovery; and repetitive training to effectively strengthen the muscles involved in stabilizing the shoulder joint, which can also effectively strengthen the muscles around the shoulder that are weakened by flaccid paralysis occurring in the early stages of stroke onset. After a stroke, weakness in the shoulder joint area and problems with joint coordination and timing of movement lead to abnormal flexion synergies in upper limb functional movements. This results in a decrease in the active joint range of motion and worsening of muscle weakness over time owing to the loss of muscle contraction opportunities [33,34]. The sling suspension system adjusted the gravity applied to the shoulder joint according to the muscle strength of the participant, enabling active muscle contraction of shoulder joint flexion, extension, abduction, adduction, external rotation, and internal rotation. Strengthening of the muscles involved in shoulder joint stabilization is thought to occur gradually through continuous repetitive training. When shoulder flexion was performed without weight load on the upper limb, the muscle activity of surrounding muscles, such as the deltoid, pectoralis major, supraspinatus, IST, subscapularis, and trapezius muscles, showed moderate levels of activation. While active contraction occurs in the shoulder joint, the prime mover and synergistic and stabilizing muscles are recruited together to perform cooperative contractions [35]. Therefore, inducing active muscle contractions in various directions are important for enhancing strength, because they involve muscles working together in synergy. In the present study, the SASE group adjusted the influence of gravity according to muscle strength, as previously reported, while lying down, lying on their side, or sitting. Active muscle contractions were repeatedly induced while gradually increasing the weight load using the position of the harness point and sandbags, so participants were able to exercise selective movements to their maximum range. We concluded that the strength of the muscles related to movement effectively increased in participants performing active exercises compared to those undergoing passive exercises through the motorized upper limb exercise device. Criswell [36] reported that the deltoid, IST, and teres major muscles are primarily engaged during shoulder abduction movements and that as joint range of motion decreases, muscle activity also decreases. In the present study, we considered that the weakness of the shoulder abductor muscles and the decrease in joint range of motion in patients with stroke made it difficult to perform precise abduction movements, leading to compensatory actions where the surrounding muscles are excessively utilized. As a result, abductor muscle strengthening occurs to a lesser extent.

Upper limb function has the lowest recovery rate during motor function recovery after stroke and poor recovery of upper limb function is recognized as a significant factor hindering independent living [37,38]. Paci et al. [39] investigated the relationship between upper limb function and shoulder subluxation in 107 patients with acute stroke and found a direct causal relationship between upper limb function and subluxation. The authors suggested that subluxation affects upper limb function. Currently, various treatment methods are used to recover upper limb function in patients with stroke. These include mirror therapy, autogenic relaxation training, EMG biofeedback, proprioceptive neuromuscular facilitation (PNF), and Bobath therapy [40]. Krabben et al. [41] reported improvement in upper limb function after applying upper limb training three times a week for 6 weeks using slings and springs on the wrist and elbow joints to compensate for gravity during involuntary flexion synergy in patients with hemiparesis poststroke.

This study used the FMA and MFT to evaluate upper limb function. The FMA score in the SASE group increased by 6.43 from 13.29 before intervention to 19.71 after intervention, while in the MUEE group, it increased by 4.07 from 9.64 before intervention to 13.71 after intervention. The MFT score in the SASE group increased by 4.5 from 6.57 before intervention to 11.07 after intervention, while in the MUEE group, it increased by 2.86 from 10.79 before intervention to 13.64 after intervention. Upper limb function was significantly different between the time–group interaction effect. Our results proved statistically significant, but only 60% of the minimal clinically important distance (MCID) was achieved. MCID of FMA reported 9 to 10 points for subacute stroke [42]. Upper limb function requires sufficient strength and neuromuscular control to generate functional movements against gravity as it involves a combination of lifting and reaching actions. Zorowitz [43] reported that upper limb function improves as the degree of shoulder subluxation decreases in stroke patients. This finding was based on a study investigating the relationship between the degree of subluxation and upper limb function in patients with stroke by comparing the affected and unaffected shoulders. The improvement in the upper limb function observed after active shoulder joint exercises using the sling suspension system in this study is consistent with previous findings.

However, this study has several limitations. First, the small sample size must be considered. The experiment involved only 28 patients among those receiving treatment at a rehabilitation hospital. Therefore, generalization of these findings to all patients with stroke should be approached with caution. Furthermore, due to the lack of a follow-up study after the 4-week intervention period, long-term maintenance of the effects could not be assessed. Additionally, only patients with a muscle strength grade of poor or worse were included in the study, which means that patients with severe movement impairment below the fair level were not eligible for inclusion.

Future research should address these limitations and studies with larger sample sizes that involve patients with a wider range of muscle strength levels, including those with severe movement impairment, and with longer follow-up periods are required.

## 5. Conclusions

This study investigated the effects of sling-suspension-based system active shoulder joint exercises on subluxation, pain, muscle strength, and upper limb function in patients with subacute stroke. Although future work is needed to optimize sling-suspension-based active shoulder joint exercises, the current study findings show decreased subluxation distance and pain, strengthening of the shoulder flexors, abductors, and external rotators, and improved upper limb function. These results suggest that sling-suspension-based active shoulder exercise can be used as an effective training method for rehabilitating subacute stroke patients.

## Figures and Tables

**Table 1:** General characteristics of participants (*n* = 28).

Characteristics	SASE Group (*n* = 14)	MUEE Group (*n* = 14)	X^2^/*t*(*p*)
Sex (M/F)	8/6	5/9	1.292(0.256)
Age (years)	59.0(17.83) ^a^	64.64(14.41)	0.921(0.366)
Height (cm)	164.57(8.63)	163.64(11.43)	0.242(0.810)
Weight (kg)	63.71(11.11)	61.98(10.51)	0.425(0.675)
Onset period (months)	4.21(0.89)	3.787(0.801)	0.334(0.193)
Lesion sites (right/left)	6/8	5/9	0.150(0.699)
Stroke type (ischemic/hemorrhagic)	9/5	8/6	0.150(0.699)

^a^ M(SD); SASE = sling-suspension-based system active shoulder joint exercise; MUEE = motorized upper extremity exercise; MMSE-K = Korean version of the mini mental state examination; MAS = modified Ashworth scale.

**Table 2:** Changes in shoulder joint subluxation (*n* = 28).

Parameters		SASE Group (*n* = 14)	MUEE Group (*n* = 14)	t(*p*)	Time *F*(*p*)	Group *F*(*p*)	Time–Group *F*(*p*)
Subluxation (mm)	Pretest	44.59(9.27) ^a^	40.03(9.28)	1.300(0.205)	49.578 (0.000)	0.729 (0.401)	6.025(0.021)
	Post-test	36.97(6.21)	36.35(8.03)				
	Mean difference	−7.61(5.66)	−3.68(1.98)	2.313(0.038)			
	t(*p*)	−5.030(0.000)	−6.943(0.000)				

^a^ M(SD); SASE = sling-suspension-based system active shoulder joint exercise; MUEE = motorized upper extremity exercise.

**Table 3:** Changes in pain (*n* = 28).

Parameters		SASE Group (*n* = 14)	MUEE Group (*n* = 14)	t(*p*)	Time *F*(*p*)	Group *F*(*p*)	Time–Group *F*(*p*)
Splenius (kgf)	Pretest	3.92(1.22) ^a^	3.85(0.74)	0.163(0.872)	384.345(0.000)	0.531(0.473)	7.470(0.011)
	Post-test	5.84(1.42)	5.31(0.90)				
	Mean difference	1.92(0.43)	1.45(0.48)	−2.386(0.033)			
	t(*p*)	16.889(0.000)	11.246(0.000)				
UTZ (kgf)	Pretest	5.30(1.60)	5.36(1.16)	−0.125(0.902)	323.720(0.000)	0.312(0.581)	9.623(0.005)
	Post-test	7.74(1.56)	7.08(1.33)				
	Mean difference	2.44(0.76)	1.72(0.42)	−3.812(0.022)			
	t(*p*)	12.035(0.000)	15.460(0.000)				
IST (kgf)	Pretest	4.27(1.39)	3.85(0.96)	0.913(0.370)	451.991(0.000)	1.088(0.307)	1.123(0.299)
	Post-test	6.06(1.55)	5.47(1.13)				
	Mean difference	1.79(0.45)	1.62(0.39)	−1.652(0.112)			
	t(*p*)	14.763(0.000)	15.428(0.000)				

^a^ M(SD); SASE = sling-suspension-based system active shoulder joint exercise; MUEE= motorized upper extremity exercise; UTZ = upper trapezius; IST = infraspinatus.

**Table 4:** Changes in the muscle strength of the shoulder joint (*n* = 28).

Parameters		SASE Group (*n* = 14)	MUEE Group (*n* = 14)	t(*p*)	Time *F*(*p*)	Group *F*(*p*)	Time–Group *F*(*p*)
Flexors (kg)	Pretest	2.17(0.74) ^a^	2.23(0.48)	−0.273(0.787)	236.665(0.000)	0.767(0.389)	13.211(0.001)
	Post-test	3.78(1.08)	3.23(0.63)				
	Mean difference	1.61(0.57)	0.99(0.26)	−3.271(0.006)			
	t(*p*)	10.469(0.000)	14.047(0.000)				
Extensors (kg)	Pretest	2.46(1.08)	2.52(0.63)	−0.192(0.849)	179.055(0.000)	0.048(0.829)	1.930(0.177)
	Post-test	4.03(1.52)	3.80(0.88)				
	Mean difference	1.58(0.63)	1.28(0.50)	−1.799(0.095)			
	t(*p*)	9.372(0.000)	9.461(0.000)				
Abductors (kg)	Pretest	2.22(0.72)	2.30(0.42)	−0.319(0.752)	128.769(0.000)	0.346(0.561)	4.974(0.035)
	Post-test	3.70(1.22)	3.28(0.72)				
	Mean difference	1.48(0.70)	0.99(0.41)	−2.289(0.039)			
	t(*p*)	7.883(0.000)	8.971(0.000)				
Adductors (kg)	Pretest	2.80(1.01)	3.01(0.68)	−0.633(0.523)	170.356(0.000)	0.104(0.749)	0.393(0.536)
	Post-test	4.43(1.48)	4.49(1.18)				
	Mean difference	1.63(0.63)	1.48(0.63)	−0.751(0.466)			
	t(*p*)	9.703(0.000)	8.758(0.000)				
External rotators (kg)	Pretest	1.73(0.70)	1.83(0.36)	−0.474(0.640)	125.739(0.000)	0.394(0.536)	9.674(0.004)
	Post-test	2.98(1.14)	2.54(0.61)				
	Mean difference	1.25(0.50)	0.70(0.42)	−5.378(0.000)			
	t(*p*)	9.347(0.000)	6.305(0.000)				
Internal rotators (kg)	Pretest	2.42(0.93)	2.91(0.74)	−1.526(0.139)	232.829(0.000)	1.591(0.218)	0.010(0.923)
	Post-test	4.10(1.21)	4.56(1.19)				
	Mean difference	1.68(0.46)	1.66(0.67)	−0.094(0.927)			
	t(*p*)	13.524(0.000)	9.208(0.000)				

^a^ M(SD); SASE = sling-suspension-based system active shoulder joint exercise; MUEE = motorized upper extremity exercise.

**Table 5:** Changes in the upper limb function (*n* = 28).

Parameters		SASE Group (*n* = 14)	MUEE Group (*n* = 14)	t(*p*)	Time *F*(*p*)	Group *F*(*p*)	Time–Group *F*(*p*)
FMA (score)	Pretest	13.29(6.44) ^a^	9.64(3.36)	1.876(0.076)	262.785(0.000)	5.805(0.023)	13.243(0.001)
	Post-test	19.71(6.74)	13.71(4.10)				
	Mean difference	6.43(1.95)	4.07(1.44)	−3.100(0.008)			
	t(*p*)	12.336(0.000)	10.585(0.000)				
MFT (score)	Pretest	6.57(5.88)	10.79(7.68)	−1.631(0.116)	647.498(0.000)	1.631(0.212)	32.286(0.000)
	Post-test	11.07(6.32)	13.64(7.99)				
	Mean difference	4.50(0.76)	2.86(0.77)	−6.618(0.000)			
	t(*p*)	22.168(0.000)	13.878(0.000)				

^a^ M(SD); SASE = sling-suspension-based system active shoulder joint exercise; MUEE = motorized upper extremity exercise; FMA = Fugl–Mayer Assessment; MFT = Manual Function Test.

## Data Availability

Data are contained within the article.
